# Accessible Tutoring Platform Using Audio-Tactile Graphics Adapted for Visually Impaired People

**DOI:** 10.3390/s22228753

**Published:** 2022-11-12

**Authors:** Michał Maćkowski, Piotr Brzoza

**Affiliations:** Department of Distributed Systems and Informatic Devices, Silesian University of Technology, 44-100 Gliwice, Poland

**Keywords:** assistive technology, audio-tactile graphics, education, tutoring system, multimodal interfaces, touch interface, people with blindness

## Abstract

One of the problems faced by people with blindness is access to materials presented in graphical form. There are many alternative forms of providing such information, but they are very often ineffective or have certain limitations. The development of mobile devices and touch sensors enabled the development of new tools to support such people. This study presents a solution called an accessible tutoring platform, using audio-tactile graphics for people with blindness. We aimed to research the influence of the developed platform for the alternative presentation of graphics information on better memorizing, recognizing, and learning. Another goal of the research was to verify the effectiveness of the proposed method for the alternative presentation of audio-tactile graphics. The effectiveness of the proposed solution was verified quantitatively and qualitatively on two groups of blind students from primary and secondary schools with the use of a developed platform and prepared materials for learning mathematics. The obtained research results show that the proposed method of verifying students’ knowledge and auto-selecting exercises with adapted audio description positively influences the improvement of learning effectiveness.

## 1. Introduction

Currently, the growth of information presented in graphic form is noticeable higher than it used to be. It is mainly due to the development of internet services, commonly used mobile phones, and mobile applications. However, people with sight problems cannot use this type of information, or access is very limited. Undoubtedly, graphic information dominates chiefly in science education, such as in Math, Physics, Chemistry, and Engineering subjects.

When it comes to the education of people with blindness, it is based on applying screen readers and speech synthesizers to read aloud educational texts. However, this technique becomes ineffective when we want to present a piece of information in graphic form. Only very basic pictures can be described in text form and read aloud by a speech synthesizer.

An alternative attitude to solve this problem can be using tactile graphics, where a user, through touch perception, is able to obtain some information about the picture [1,2,3]. Tactile pictures include raised lines and elevated surfaces that are detectable under the fingers. Such pictures must be prepared in an appropriate way so that a blind user can recognize them and interpret their content. This method is frequently used in braille books. Nevertheless, it fails in the case of complicated pictures containing various elements. Using only touch sense to present information is insufficient to substitute sense of sight and interpret its content correctly.

The popular solution is applying braille labels for elements in the tactile pictures and corresponding descriptions on the same or separate braille sheets of paper [4,5]. However, by continuously taking hands away from the picture and placing them on paper with braille descriptions to obtain information about the particular graphic element, the user loses the context of the touched item. Then the user must start this operation from the beginning. It results in a poor understanding of the material and, at the same time, leads to cognitive overload [6].

This study presents a solution called an accessible tutoring platform, using audio-tactile graphics for people with blindness. It aimed to research the influence of the developed platform for the alternative presentation of graphics information on better memorizing, recognizing, and learning. Another goal of the research was to verify the effectiveness of the proposed method for the alternative presentation of audio-tactile graphics. Furthermore, it considered a quantitative and qualitative assessment of the learning progress of blind students using the presented platform. The study also analyzed the influence of the proposed method of exercise auto-selection and context information adequacy to meet the needs of students with blindness from primary and secondary schools.

The platform is characterized in detail in Section 3, where it presents particular elements and their functionalities. The next sections present the research group, methodology, and research results. The research was conducted in Education Centre for the Blind Students on students groups from primary and secondary schools.

## 2. Related Works

Based on the literature review, we notice many research works on alternative methods of presenting graphic information to people with blindness, especially in interactive audio-tactile form. The analyses of the results presented in the reviewed papers indicate several methods for element identification of a picture that a blind user touches to read their verbal description aloud.

The first attitude uses dedicated touch tablets, e.g., the Talking Tactile Tablet produced by Touch Graphis and the IVEO by ViewPlus2, connected to the computer. Moreover, the companies provide dedicated applications for preparing pictures with audio descriptions and a browser for interactive audio-tactile presentations of prepared graphics. The mentioned devices were used in many scientific works where the authors focused on applying them in the educational process [7,8,9,10,11]. Though the above solution seems beneficial, its high price and low mobility may be considered as limitations. What is more, the large size of the device allows us to acquire a convenient presentation of tactile pictures; nonetheless, it is heavy and hard to carry from one place to another.

Another attitude presents applying a method of visual tracking of activities performed by the user (movement of the fingers on a tactile picture) using cameras and dedicated image recognition software. The elements in the picture touched by the user have special labels containing QR codes, and they are identified using artificial intelligence (AI) methods for image processing [12,13,14,15]. It provides us with the opportunity to hear the audio description of the picture content. However, these solutions have some minor negative points. First, the system operation depends on the light condition in the classroom. Next, the quality of the cameras, not to mention the complexity of the picture recognition process, need improving. An extension of this idea is research into the use of augmented reality (AR) to provide contextual information about the touched elements [16,17].

As a reasonably innovative and prototypical technique, we can consider applying dynamic haptic displays for the presentation of graphic elements. The main function of the matrix is to present an excerpt of a picture from the computer screen using dynamically pushed pins (taxels) [18]. Examples of such devices are DotView from KGS Corporation, the Graphic Window by Handytech, and the Graphiti Interactive Tactile Graphic Display by ORBIT Research. There is also research into the identification of elements on a dynamically presented tactile picture (the areas touched by the user’s fingers are immediately detected). Furthermore, papers [19,20,21] describe applying such devices for map presentation and the navigation of a blind user inside a building. Undoubtedly, the very high price (from EUR 5 to 20 thousand) of these devices considerably limits their common application.

The rapid development of mobile devices (phones and tablets) and their widespread availability has encouraged scientists to conduct research on applying these devices for the interactive presentation of audio-tactile graphics. Tactile pictures are placed on a tablet screen supplied with a dedicated application that detects the elements of the picture that the user is touching. Next, it provides audio information about the elements. Such functionality is possible by supplying the devices with a capacitive touch screen. Due to this, the touch of an element is detected on the device screen through a sheet of paper. Tactile pictures can also be printed with braille embosser or on specialized swell touch paper, or printed via 3D technology. The advantage of the last technique is the possibility of differentiating the height of printed elements significantly. However, it is time-consuming and requires printers with a large working area (A4 size). Two papers [22,23] depict using 3D prints for presenting maps and building plans as audio-tactile graphics. The authors of these papers tested multiple types of graphics and obtained evidence that visual augmentation may offer advantages for the exploration of tactile graphics.

The idea of using the previously mentioned solution appears valuable for education, especially for science subjects where the presentation of information in a graphic form takes place frequently. The authors of one paper [24] considered problems with access to graphics at school from the point of view of both teachers and students. The authors also concluded that we need to consider experience, price, and time for picture adaptation to prepare tactile pictures. For students, on the other hand, important factors are the ease of using the device and application, the ease of interpreting the tactile pictures, and the audio description for touched elements. As the research results indicate, this method helps the student make fast progress and improves their ability to acquire knowledge and skills in a particular subject. Much of the research in this area has been described and assessed in the paper of [25].

The former research conducted by the author focused on the assessment of touch perception among people with blindness and methods of alternative audio presentation of math formulae [26,27,28]. Within the previous research, the authors developed an intelligent platform for math learning that encompassed math analysis at an academic level. This platform has been used for a few years by both sighted students and those with blindness. We developed rules for the interactive reading of math formulae in the Polish language and their structures for the platform [29,30]. The cooperation with educational centers for students with blindness and the gained experience motivated the authors to research alternative methods concerning the presentation of graphics to blind people for educational use.

The present study’s contribution is the development of a multimodal method of alternative presentation of graphic information in the form of interactive audio-tactile graphics. The research on the effectiveness of the developed method was verified in the process of adapted mathematics teaching for people with blindness using the developed platform. We also propose audio-tactile descriptions with different levels of detail, considering the increasing number of terms occurring in audio descriptions adapted to the students’ knowledge and skill level from selected math areas. The effectiveness of this solution was verified quantitatively and qualitatively on two groups of blind students from primary and secondary schools.

## 3. Designed Accessible Tutoring Platform

The primary step was to analyze many scientific research projects regarding the alternative presentation of graphics to blind people. Based on the literature review and the preliminary research on touch perception among blind people, the decision was made to design and implement the platform using interactive audio-tactile graphics adjusted to the needs of students with blindness. Before the platform was developed, the researchers conducted numerous interviews and consultations with students with blindness and their teachers. Based on the consultations, the following suggestions from students and teachers were collected, which were later taken into account when designing our platform:

1. The interface of the mobile application should be easy to use and include the audio-touch interface.
2. To use the mobile application, simple touch gestures such as single and multiple taps on the screen should be used.
3. The mobile application should work well with the system screen reader or use the self-voicing mode.
4. The mobile device should enable easy placement of the tactile picture on the screen in a specific position. The tactile picture should coincide with the picture displayed on the screen. The sheet of paper should be fixed to the device so that it does not move while moving the fingers on the pictures.
5. The application should enable solving tests corresponding to the exercises using the voice interface and the automatic function of sending test results to the server.
6. The server application should run in a web browser, e.g., Mozilla Firefox or Google Chrome.
7. The server application should enable the upload of previously prepared pictures in SVG format and addition of audio descriptions for image elements.
8. The platform should enable the preparation of exercises along with instructions, pictures, and tests at various levels of difficulty.
9. The platform should enable the grouping of exercises into subjects and topics, and enroll students in a selected course.
10. Using the platform, the teacher should be able to view the exercises and test results produced by the students.

The developed platform was designed according to web accessibility standards and guidelines [31]. While designing the platform, the study assumed that the system consisted of a server application with a web interface coupled with the corresponding mobile application for a student used for presenting audio-tactile pictures.

### 3.1. Server with a Web Application

The server included in the platform is responsible for preparing and sounding graphic materials and sharing chosen exercises with particular students or groups. The web platform also allows one to prepare and distribute tests assigned to specific exercises and pictures for students to solve on mobile devices. In addition, the web platform logs information about the learning progress of individual students. It allows teachers to assess the level of knowledge of the student and their progress. The teacher can also analyze the mistakes made by a group of students for a selected category of exercises, which enables the identification of common mistakes at the level of the whole group. Moreover, they also can modify the descriptions of the pictures and questions included in the tests. Educational materials are grouped in the platform according to courses and school classes. Each student is assigned to a specific course and exercises that should be solved.

The exercises are also grouped according to the category of exercises, e.g., properties of function based on its graph, properties of geometric figures, graphical representations of system of equations, etc. For each category of exercises, the platform provides the opportunity to define the class of mistakes possible to make when solving the exercise, e.g., zero places and function domain. Based on this information, the platform intelligently analyzes the history and frequency of mistakes made by a student and, in consequence, selects another exercise.

**Figure 1:**
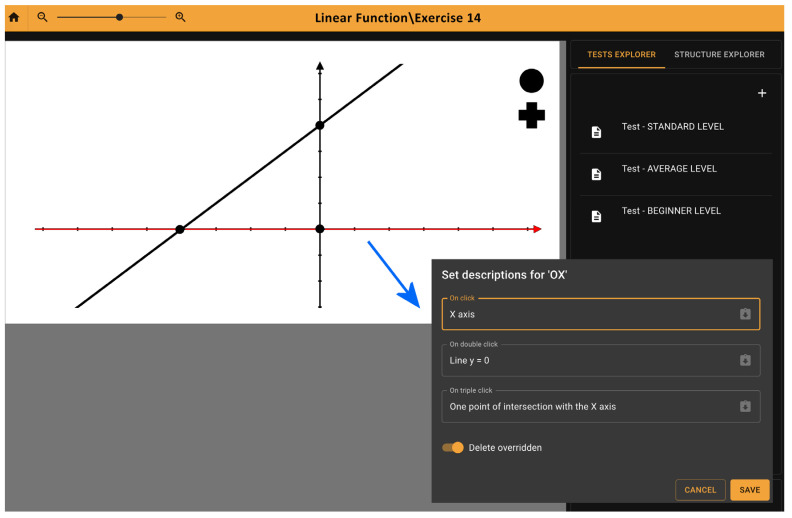
The interface of the web application while preparing audio descriptions for the selected picture element (X axis marked in red color).

Figure 1 presents the interface of a web application while preparing audio descriptions to the tactile image. First, we prepare a picture in any SVG editor. Next, we import it into our platform. Here, each graphic element of a picture can be supplied with an audio description by adding text information read aloud after single, double, or triple taps on a particular element of a picture. We can also export previously prepared pictures from the platform to pdf file and then print them on a braille printer or swell touch paper.

### 3.2. Mobile Application for Interactive Presentation of Audio-Tactile Graphics

**Figure 2:**
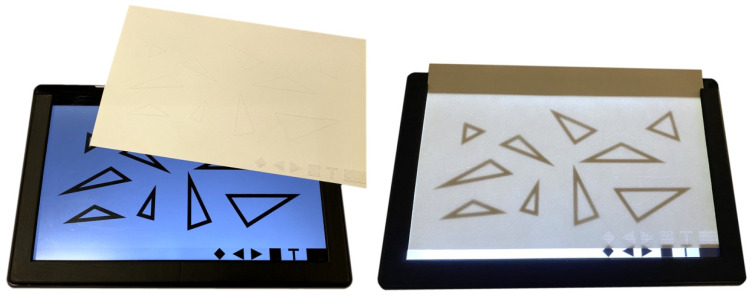
A tactile picture containing a sample image for learning to recognize the properties of triangles, placed in a frame on the tablet screen—test bench.

The mobile application using audio-tactile graphics for blind students’ education is dedicated to the Android system. It uses tablets with a large standard screen diagonal (approx. 13 inches), which is very convenient when presenting the content of tactile pictures in A4 paper size. During the implementation of the application, it was important to take into account the mobile accessibility rules defined at W3C [32]. The main assumption is that the application displays an exercise (a picture) on a tablet screen to the corresponding tactile picture printed on paper. The student must place the paper print on the tablet screen so that the tactile picture covers the picture displayed on the screen. To simplify this process, a frame was printed via 3D technology, which helped to hold the tactile picture in a still position. The tests confirmed that it is possible to recognize the gestures in tablets made by a student through the paper placed on the screen (paper print is not an obstacle in recognizing the gestures). Figure 2 shows a tactile picture with an exemplary image for recognizing the properties of triangles. It is placed in the tablet frame. This test bench was used during the research.

Moreover, the mobile application also gained an audio-tactile easy-to-use interface (bottom-right corner of the screen—see Figure 2). The interface consists of six buttons placed horizontally, and enables easy navigation in the application menu, which is read aloud. Each button has its own dedicated function:

- Menu and test navigation;
- Back—undo;
- Next;
- Confirm;
- Choose a test;
- Application settings.

The mobile application also enables us to adjust personalized settings to the needs of a particular student, i.e., the thickness of lines displayed on the screen. Considering conducted research and the research results included in papers [33,34,35], we can say that the line thickness is an essential parameter depending on the particular perception skills of an individual student. With too-thin lines, a student, while touching an element on a tactile picture, will not be able to touch the corresponding graphic element on the tablet screen. In consequence, the application will not read the audio description.

The student can also choose the time interval between two sequential taps (from 250 to 750 ms) to adjust the speed of recognized gestures to their needs. The application uses the standard Text To Speech synthesizer available on the Android system. The student can thus adjust the speech parameters (speed, volume, pitch) according to their preferences.

Each exercise has corresponding tests of multiple-choice questions and answers. The mobile application enables the students to solve the tests interactively and send the results to be assessed to the server.

Information collected on the server about the exercises being solved, learning progress, and times of solving individual exercises allowed the implementation of the function of intelligent selection of exercises, based on the history of mistakes made by a particular student. Moreover, this information is used to modify the audio description of elements in the picture adequately to the level and experience of a student. This means that a less-skilled student or beginner provides more information about touched elements and their properties (hints, definitions, examples).

## 4. Materials and Methods

### 4.1. Research Group

The participants in the tests were secondary school students (first group) aged 15–20 years old with a high level of blindness. The group consisted of 20 students (12 boys and 8 girls). Each of the students was familiarized with the required material. In the presented group, 16 people were completely blind, and 4 were partially sighted.

The second group consisted of primary school, 4th–6th grade, students. There were 24 students, of which 18 were blind and 6 were visually impaired. In terms of gender, the group consisted of 13 boys and 11 girls. The whole group possessed average knowledge of and experience in mathematics. The participants in the research group were chosen after consultations with their math teachers. None of them had other disabilities, especially motor, hearing, or mental.

### 4.2. Research Materials

Two sets of materials were prepared for the research. They were used to assess the level of perception, recognition, memorization, and usability of the developed methods for the presentation of audio-tactile graphics.

- The first set of materials included tactile pictures with braille descriptions, similar to those found in braille books. Each value element of a picture had a braille label (e.g., T1, C2, etc.), and on a separate sheet of paper were braille descriptions corresponding to the particular label, e.g., T1 and right-angled triangle, respectively.
- The second group encompassed interactive audio-tactile graphics. Each essential element of the picture had a verbal description, read after tapping on it. Moreover, 1 to 3 descriptions were prepared for each picture element. The type of description depended on the number of taps:
  ○ 1 Tap—simple description, e.g., an element name;
  ○ 2 Taps—detailed description, e.g., includes information about the object features, such as size, angle, field;
  ○ 3 Taps—advanced description, e.g., includes definitions of terms, hints, and examples.

**Figure 3:**
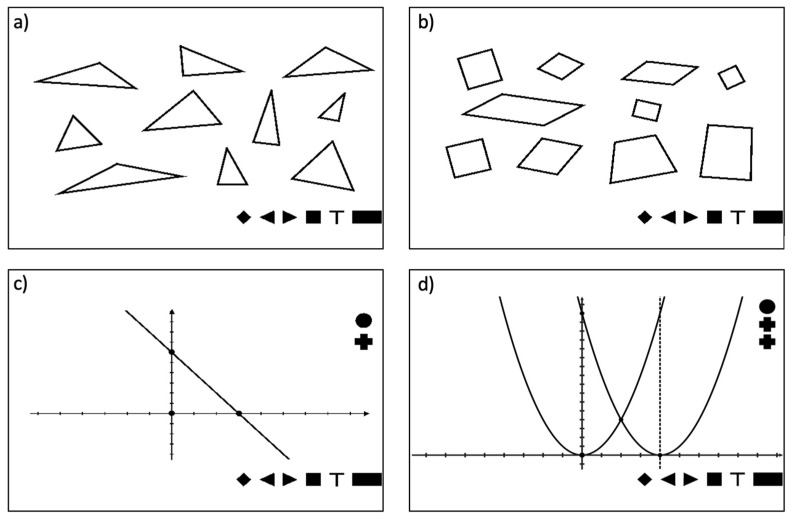
Examples of pictures for exercises used for research in primary school: (**a**) recognizing the properties of triangles; (**b**) recognizing the properties of quadrilaterals (also in secondary school); (**c**) linear function; (**d**) quadratic function.

For each group of materials, a set of exercises in the form of a test with a list of questions and multiple-choice answers was elaborated. There was a set of 20 different pictures prepared for primary school students, containing geometric figures such as triangles, quadrilaterals, circles, ellipses, and polygons. These materials were used to evaluate the effectiveness of recognizing shapes, sizes, and properties of geometric figures. Figure 3a,b show exemplary pictures used during the research.

The materials for secondary school students included 40 exercises on the properties of functions (linear, quadratic, homographic, exponential, and trigonometric functions)—Figure 3c,d. These materials were used to test the effectiveness of recognizing the properties of functions based on their graphs, e.g., domain, set of values, zero places, monotonicity, maximum, minimum, and periodicity.

Each exercise was prepared with three different levels of complexity, i.e., Standard, Average, and Beginner, and a corresponding audio description for each picture element. For the Beginner exercise level, the audio descriptions are more detailed, and contain additional information about picture elements and their features (Table 1 column “Element description for Beginner exercise”). In contrast, the Standard level exercises include only the necessary audio descriptions that are needed to resolve the exercise (Table 1 column “Element description for standard exercise”). Table 1 presents audio descriptions for image elements of the example exercise for linear function. Appendix A, also contains Table A1, presenting 3 levels of this exercise complexity: Standard, Average, and Beginner.

Depending on the number of taps of the element (1, 2, or 3), an appropriate audio description of the element is read. The texts of the audio descriptions prepared for individual elements of the image are presented in Table 1. The symbols *, **, and *** in Table 1 refer to audio descriptions assigned to a given element of the tactile image when performing a single, double, or triple tap, respectively.

### 4.3. Research Methodology

Before the research commenced, all participants were familiarized with the pictures with braille descriptions. They were also shown how to use the application for interactive presentation of audio-tactile graphics, and introduced exemplary images containing audio descriptions for particular elements.

The conducted research concerned two aspects. First, we examined the effectiveness of learning with the platform compared to traditional learning using braille books with tactile pictures. In the next stage of the research, we focused on the effectiveness of learning with an intelligent mode in the platform (automatic assessment of the student’s knowledge and selection of subsequent exercises to be solved). The effectiveness of learning with the platform was verified by measuring the number of mistakes made by the students when solving exercises of recognizing geometric figures and recognizing the properties of functions based on their graphs. We formulated the research hypothesis that “learning with the developed platform and the method of automatic selection of exercises adapted to the level of student’s knowledge permits more effective learning and fewer mistakes when solving exercises”. A more detailed description of the research carried out on the group of primary and secondary school students is presented below.

#### 4.3.1. Research with Participants from Primary School (PS)

In the first phase (Experiment PS1), the evaluation concerned the recognition of geometric figures and the number of mistakes made by students. The obtained results in recognizing shapes, sizes, and properties of the figures using traditional materials (only a tactile picture with braille description) were contrasted with the results obtained when the developed platform for interactive audio-tactile graphics was used.

The students had to answer the test questions by identifying the properties of the figures in the picture. Each of the participants solved 5 exercises for a particular type of figure. Each exercise contained 10 different figures on the corresponding tactile picture.

#### 4.3.2. Research with Participants from Secondary School (SS)

In the first phase (Experiment SS1,) the evaluation concerned the times for recognizing the properties of the function and the number of mistakes made in interpreting the function properties using traditional materials (only a tactile picture with braille description) and when the developed platform for interactive audio-tactile graphics was used.

The student had to answer the test questions by identifying the function properties in the picture. Each of the participants solved 5 exercises for a particular type of function.

In both tests, PS1 and SS1, the results were grouped for each user and for individual types of mathematical functions and geometric figures. The research at both schools was conducted for 2 weeks, and a single weekly session lasted about 2 h. Moreover, the process of solving the exercises was supervised by math teachers of the blind students, who wrote down their own observations and comments on the users.

The second phase of the research was conducted both in primary and secondary schools (Experiments PS2 and SS2) and started in the 3rd week of the research. Here, the idea was to adjust the description of elements in the picture (detail and advanced) based on the history of mistakes made by a student and their knowledge. The platform, on the basis of collected information (mistakes made and times for answering the question), qualified a student to a particular group, i.e., beginner, average, and advanced users, and proposed subsequent exercises adapted to the student’s knowledge level (Standard, Average, or Beginner exercises). Beginners or those with problems in mastering the material (understanding and memorizing) received a wider variety and more precise descriptions of the elements in the picture.

The mode described above was named the intelligent learning mode with the platform. While working in this mode, the student solves 3 exercises from the selected category at the standard level. If the number of mistakes made by the student from the same category is greater than 1, the platform classifies the student into a group with a lower level of experience and allows him/her to solve exercises from the same category with a lower level of complexity and with an increased number of descriptions of picture elements. The research assumed that the possibility of making a random mistake when solving exercises from the same category does not affect the assessment of the student’s level of knowledge.

**Figure 4:**
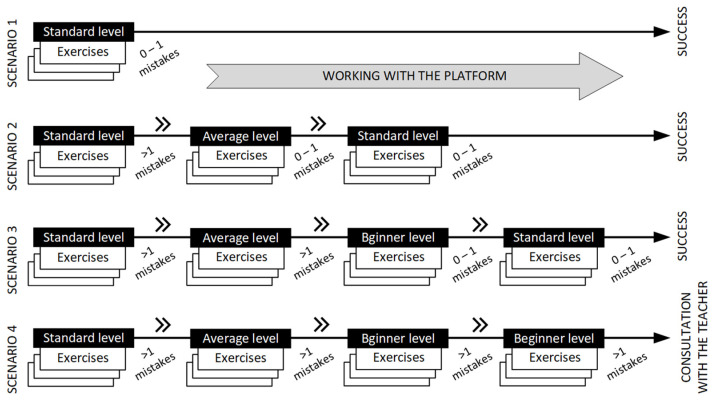
Graphs of students learning processes using the developed platform for different scenarios.

Figure 4 presents the example graphs of students learning processes using the developed platform for different scenarios. Each student starts working with the platform by solving 3 exercises at a standard level, covering exercises with the same class of mistakes. Based on the number of mistakes made, the platform selects another set of exercises at a lower level of difficulty; for example, a student who made more than 1 mistake in a given class then solves the exercise at an average level (Figure 4 scenario 2). If the student masters the material at this level, the platform again selects a set of exercises at the standard level. However, if the student still makes a significant number of mistakes at the secondary level, the platform offers him/her the option to solve the Beginner-level exercises (Figure 4 scenario 3). Scenario 4 shows a situation in which, despite working several times with the platform at the Beginner level, the student still makes a large number of mistakes and, in such a situation, is directed to consult the teacher.

In the case of the analysis of function properties based on the graph, the student was asked about the following features: domain, set of values, zero places, monotonicity, maximum, minimum, and y-intercept. For the properties of the geometric figures, the research referred to the comparison of the area of the figures and their properties based on their shape, i.e., correct recognition of triangles (equilateral, isosceles, scalene, acute, right-angled, obtuse) and quadrilaterals (square, rectangle, rhombus, parallelogram, and trapezoid).

At the end of each session, participants of the research answered a series of questions about the usability and satisfaction of using the developed platform and the intelligibility of audio descriptions assigned to individual image elements. Each question was rated on a 1–5 Likert scale (1—worst, 2—bad, 3—average, 4—good, 5—best). As part of the questionary, we asked the following questions:

1. Q1: How do you rate the ease of learning with the use of the developed platform?
2. Q2: Were the audio descriptions of the image elements understandable and sufficiently accurate?
3. Q3: Was the proposed interface for operating the mobile application ergonomic and comfortable?
4. Q4: How do you rate the method of solving tests in the exercises?
5. Q5: How do you rate the platform mode of automatic adjusting the exercise level and contextual information according to your knowledge and experience?

## 5. Results

### 5.1. Research Results for Primary School

**Figure 5:**
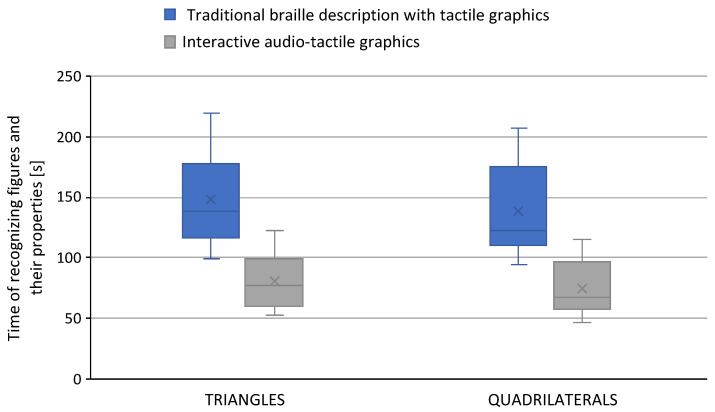
Comparison of figure recognition times and their properties (triangles and quadrilaterals) with the use of only braille descriptions and interactive audio descriptions (in the platform).

In the first phase of the research (Experiment PS1), the study focused on testing the times for recognizing the geometrical figures and the number of mistakes in interpreting the properties of the figures. Figure 5 shows the distribution of the obtained times for figure recognition (triangles and quadrilaterals) and the properties for materials containing only braille descriptions and interactive audio-tactile materials (using the developed platform). Figure 5 presents a total time of recognition for 10 figures printed on a single sheet of A4 braille paper.

**Figure 6:**
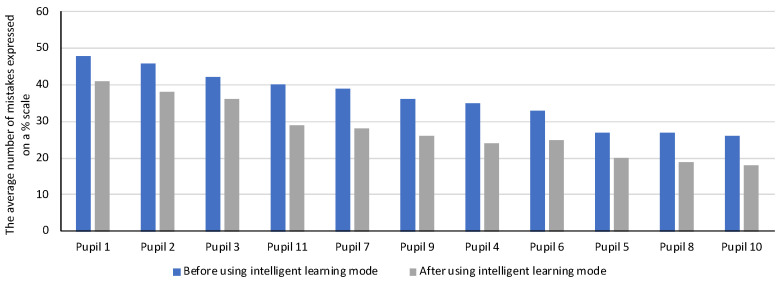
The average number of mistakes made by the pupils before and after using intelligent learning mode in the platform.

After the 2-week first phase of the research at the primary school, which referred to recognizing geometric figures and their properties, the system, based on analyzed mistakes made and the time taken to solve exercises by users, classified 11 pupils out of 24 in the group having difficulties in solving this type of exercise (the average percentage level of mistakes made by a user was greater than 25%). For this reason, the system adapted the level of hints and audio descriptions to the level of students’ knowledge—providing additional information, hints, and definitions about the properties of triangles (in terms of side lengths and types of angles) and quadrilaterals. Figure 6 illustrates a summary of the number of mistakes made by each of the 11 pupils when solving exercises before and after applying the auto-audio description adjustment in the developed system. The results were sorted with decreasing number of mistakes made by pupils. When analyzing the obtained results, we can conclude that the average number of mistakes in this group decreased from 36% to 26%.

### 5.2. Research Results for Secondary School

The first phase of the research (Experiment SS1) covered testing the times for recognition properties of the function and the number of mistakes made in interpreting its properties when the student used only braille descriptions, and then our platform, when solving the exercises. Table 2 presents a comparison of the average times for two exemplary classes of functions when identifying six function properties based on its graph.

In research SS2, conducted from the 3rd week of the tests, the system based on the history of received students’ results and the times of solving an exercise selected the descriptions intelligently to the element in the picture. In the case of the linear function, for six students, solving the exercises was problematic. In contrast, 14 participants found difficulties in solving exercises referring to quadratic functions.

Table 3 and Table 4, respectively, present the results of the average number of mistakes made by these students before and after applying the intelligent learning mode for the linear and quadratic functions.

In order to show statistically significant differences between the obtained results before and after applying the intelligent mode in the platform, the statistics for the paired (dependent) T Test were determined. Before calculating the statistics, the normality of the distribution of values in individual experiments was checked using the Shapiro–Wilk test. For the assumed significance level (α) = 0.05 in both experiments, the Shapiro–Wilk tests did not show a significance departure from the normality, thus it was assumed that the data were normally distributed. Table 5 presents the results of the T test statistic (significance level (α) = 0.05) to compare the number of mistakes made by students without and with the use of intelligent learning mode in the platform.

Results of the paired T test indicate that there is a significant large difference between before and after applying the intelligent mode in the platform. In both experiments, the *p*-value < α, thus H0 is rejected. In other words, the sample difference between the averages of after and before application is large enough to be statistically significant. Moreover, the analysis of the mean and standard deviation in both experiments shows that the average number of mistakes made before and after applying the intelligent mode in the platform is statistically significant—students made less mistakes when solving math exercises using the intelligent platform mode.

### 5.3. Questionary Research Result

**Figure 7:**
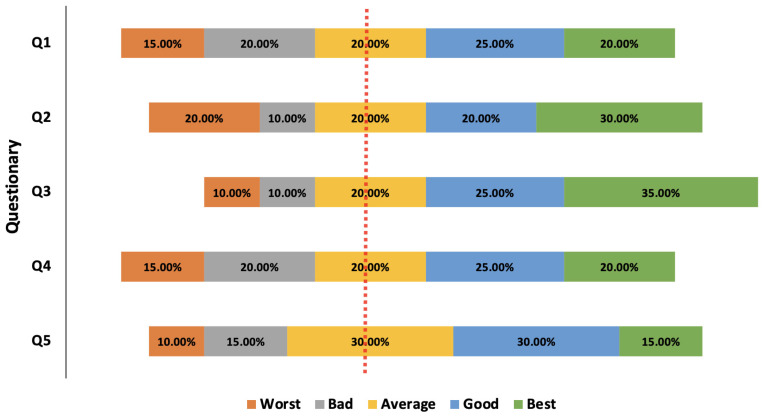
Questionary research results using the Likert scale.

The Figure 7 presents the questionary results of research participants after completing the experiments. The questionary questions are presented in Section 4.3. The results are presented as a diverging stacked bar chart for the Likert scale in normalized percentage values. The Likert scale provides a quantitative value of qualitative data obtained from the questionnaires. We can observe that more answers are on the right side of the diverging line, therefore the students positively assessed the developed platform and proposed learning method.

In Figure 7, it can be seen that in two categories, the majority of students expressed their opinions very positively. These categories concern the ergonomics of the proposed user interface of the mobile application (Q3) and the good understanding of the audio-descriptions of tactile elements on the image (Q2). In the (Q1) and (Q4) categories, positive feedback prevailed among the respondents. However, compared to other categories of questions, slightly more users assessed the method of learning and solving tests slightly negatively. In our opinion, this may be due to insufficient time spent on working with the platform and learning with the use of such multimedia tools. Moreover, the participants of the research had different levels of mathematical knowledge.

## 6. Discussion

Based on the research results, we can observe a significant differentiation in the times of solving the exercises and mistakes made between using tactile pictures with only braille descriptions and with interactive audio-tactile pictures. The traditional method for the presentation of tactile graphics, still commonly used, has numerous constraints. After identifying an element in the picture and its braille label, the student has to take their hands away from the picture and read the corresponding description on a separate braille sheet of paper. It often results in student distraction and cognitive overload, as the student has to find the required element in the picture and recognize its properties. Another disadvantage is using additional lines in the picture (with references to the text), which can disturb the correct interpretation of the picture. In the case of complex images with many elements, such additional elements (markers, guides, etc.) significantly affect the time and correctness of recognizing the properties of elements in the picture.

The results of our comparative research carried out on primary school pupils in recognizing the properties of geometric figures (triangles and quadrilaterals) presented in Figure 5 illustrate the differences in the time taken to solve exercises using the traditional method and using interactive audio-tactile graphics. In recognizing the properties of triangles and quadrilaterals, a reduction in the average time taken to solve exercises was observed, of 63 and 65 s, respectively. During the research, a reduction in the number of mistakes made using the interactive presentation method was also observed (the student touches and recognizes the selected element and at the same time receives verbal descriptions about its properties)—Figure 6.

In addition, the developed platform collects information on the number and type of mistakes and the time taken to solve specific exercises for each user. Based on this information, the system classifies the user into the appropriate group. It intelligently proposes that the user solve further exercises containing problematic issues, simultaneously increasing the amount of additional contextual audio information about the touched elements (tips/definitions/examples). It allows the student to understand a given element’s properties better and to remember them more permanently. In the subsequent phases of exercise solving, when the system assesses the improvement in the results of solving an exercise by a specific user, it classifies the user into a more advanced group, reducing, at the same time, the amount of additional contextual information.

Figure 6 summarizes the average number of mistakes made by students before and after using intelligent exercises selection and additional contextual information. In the case of primary school students, the selected group included 11 pupils, whose number of mistakes in the first phase of the study was over 25%. In this group, a decrease in the number of mistakes made when solving exercises in 3 successive weeks was observed—the average number of mistakes made decreased by 10%.

Similar research was conducted on 20 secondary school students; Table 3 and Table 4 present the results for the average number of mistakes made in determining the properties of the linear and quadratic functions based on their graphs. In the linear function, most students had no problems determining their properties, mainly due to the low complexity of the picture and the simplicity of the graph of the linear function. Only six students made more than 25% of mistakes, and in this group, additional studies were carried out to assess the impact of intelligent adaptation of the information provided. In the quadratic function exercise, an increase in the number of students committing more than 25% of mistakes was observed, which can be explained by the increased complexity of the picture (shape and direction of the parabola, axis of symmetry, etc.). The students and teachers participating in the research noted in the surveys that the complexity of the shape of the elements in the picture had a significant impact on recognizing the properties of the function.

For both the linear and quadratic functions, in subsequent studies conducted within 3–5 weeks using an intelligent selection of exercises and contextual information about elements in tactile images, a reduction in the number of mistakes made by research participants was observed, of 32% and 21%, respectively. A significant variation in results achieved by students during the research was also caused by human factors, i.e., stress, problems with focusing, tiredness, motivation, and problems with memorization.

This study has some limitations. The study lacks the quantification of the mathematical skills of students before the experiment. This procedure was performed only purposely to avoid students experiencing overload and discouragement. However, our future study intends to cover this area. Additionally, the study did not include the auto-adaptability of the presentation parameters (line thickness, line spacing) on a tablet screen. The students could choose the parameters manually, but according to our observations, the choice was not always advantageous. The current study included only the parameters based on the rules for preparing tactile images included in the Guidelines and Standards for Tactile Graphics [35]. Nonetheless, such parameters as the limited tablet screen size, resolution, and tap detection accuracy (the problem of tapping on a specified point on the tablet screen) requires additional development in this area.

Due to the cooperation of the center for the blind, it was possible to conduct the research on a relatively large group of students. The focus was on the analysis of the developed platform and the proposed method of supporting learning only in the field of mathematics. However, audio-tactile graphics can also be applied in science; therefore, the research may be extended to other areas in the future. Meanwhile, the author believes these limitations have not significantly influenced the primary outcome of the study.

## 7. Conclusions and Future Works

Using a multimodal audio-tactile user interface to present graphics information for people with sight problems contributes to a better understanding of the educational material. The developed platform not only provides information in the audio-tactile form, but also allows us to assess the level of student’s knowledge automatically during interactive exercise solving, as well as select subsequent exercises automatically and adjust the audio descriptions of the touched elements. The use of such an approach allows the students to increase their knowledge and skills and also their level of satisfaction. Sentiment analysis and NASA Task Load Index are in the plans for future work.

In addition, the web application included in the system allows us to analyze the test results of individual students and the modification of existing exercises to the specific needs of a given student.

Currently, in the developed solution, it may be problematic to identify the correct tactile pictures placed on the tablet screen according to the exercise being solved. Each tactile image contains the number and title of exercise in braille corresponding to the number of the exercise selected on the tablet. The solution to this problem may be using NFC tags or QR codes placed on the paper, which will enable their automatic recognition by the application on the user’s tablet.

In the future, the application of the platform may be extended in teaching other science subjects, such as Physics, Chemistry, Geography, and Biology, i.e., wherever the information is often provided in graphic form. Moreover, there are plans to adapt the platform to supervised remote work (distance education), where the teacher, apart from voice communication (audio channel in the application), can provide instructions to the student on an ongoing basis. Next, the teacher can observe in real time the activities and actions performed by the student (touched elements in the picture, etc.). Another goal is to consider language internationalization of the platform to other languages to conduct the research on the broader group of students.

Based on the analysis of the questionnaire conducted on a group of visually impaired students and their teachers, it can be concluded that the developed platform improves the level of tactile picture recognition and the learning process. Students very often were more willing to solve exercises using a mobile platform, arguing this with an easier understanding of the presented content in a tactile image and the possibility of independent learning without teacher supervision. There is a strong belief that applying the developed platform improves the education of students with blindness and helps to increase the level of equal opportunities in professional and social life.

## Figures and Tables

**Table 1:** Alternative audio description of the elements in the picture for 3 types of level details. (The symbols *, **, and *** in the table refer to audio descriptions assigned to a given element of the tactile image when performing a single, double, or triple tap).

Element Description for Standard Exercise	Element Description for Average Exercise	Element Description for Beginner Exercise
* Y axis * X axis * Origin of the coordinate system. * x= −4 * Point with coordinates (0, 4) (Graph of the function over the X axis) * Function y = x + 4 (Graph of the function under the X axis) * Function y = x + 4	* Y axis ** Line x = 0 * X axis ** Line y = 0 * Origin of the coordinate system. * Zero places of the function. ** x= −4 * Intersection of the function graph with the Y axis. (Graph of the function over the X axis) * Function y = x + 4 (Graph of the function under the X axis) * Function y = x + 4	* Y axis ** Line x = 0 *** One point of intersection with the Y axis * X axis ** Line y = 0 *** One point of intersection with the X axis * Origin of the coordinate system. ** Point with coordinates (0,0). * Zero places of the function. ** x= −4 *** The point with coordinates (4, 0) intersection with the X axis * Intersection of the function graph with the Y axis. ** Point with coordinates (0, 4) (Graph of the function over the X axis) * Function y = x + 4 ** The function takes positive values for x > −4 (Graph of the function under the X axis) * Function y = x + 4 ** The function takes negative values for x < −4

**Table 2:** Average time for students to complete the exercises for the two classes of functions.

	Average Time Taken to Solve the Exercises, Expressed in Seconds	
	Tactile Image with Braille Description	Audio-Tactile Image in Developed Platform
Linear function	141 (SD = 22)	45 (SD = 13)
Quadratic function	215 (SD = 23)	84 (SD = 14)

**Table 3:** Comparison of the average number of mistakes made by the student (in % scale) when solving exercises for linear functions, before and after applying the intelligent learning mode in our platform.

	The Average Number of Mistakes (in % Scale) Made by the Selected Group of Students when Solving Exercises for Linear Function		Difference in the Average Number of Mistakes in the Achieved Results (Before and After)
	Before Applying the Intelligent Mode in the Platform	After Applying the Intelligent Mode in the Platform	
Student 1	55	36	19
Student 2	40	28	12
Student 3	32	15	17
Student 4	28	14	14
Student 5	28	30	−2
Student 6	27	18	9

**Table 4:** Comparison of the average number of mistakes made by the student (in % scale) when solving exercises for quadratic functions, before and after using the intelligent learning mode in our platform.

	The Average Number of Mistakes (in % Scale) Made by the Selected Group of Students when Solving Exercises from Quadratic Function		Difference in the Average Number of Mistakes in the Achieved Results (Before and After)
	Before Applying the Intelligent Mode in the Platform	After Applying the Intelligent Mode in the Platform	
Student 1	75	53	22
Student 2	72	78	−6
Student 3	68	62	6
Student 4	68	55	13
Student 5	54	45	9
Student 6	50	40	10
Student 7	48	24	24
Student 8	46	35	11
Student 9	40	18	22
Student 10	39	15	24
Student 11	38	32	6
Student 12	35	35	0
Student 13	33	35	−2
Student 14	28	21	7

**Table 5:** Paired T Test results for the number of mistakes during solving exercises before and after using the intelligent learning mode in our platform.

	Before Applying the Intelligent Mode in the Platform	After Applying the Intelligent mode in the Platform	*p*-Value
Number of mistakes during solving exercises for linear function	M = 35, SD = 10.9	M = 23.5, SD = 9.1	0.01324
Number of mistakes during solving exercises for quadratic function	M = 49.6, SD = 15.6	M = 39.1, SD = 18	0.001477

## Data Availability

Not applicable.

## Appendix A

**Table A1:** Examples of tests with different levels of complexity for the selected linear function exercise.

Standard Exercise Level	Average Exercise Level	Beginner Exercise Level
**Question 1.** The function plot forms an angle with X axis equal (a) 30 degrees (b) 45 degrees (c) 60 degrees (d) 90 degrees **Question 2.** The function takes negative values for: (a) x > − 4 (b) x < − 4 (c) x > 4 (d) x < 4 **Question 3.** Which point belongs to the graph of the function: (a) −6, 2 (b) −6, −2 (c) −2, 6 (d) −2, −8 **Question 4.** The graph of the function with the axes of the coordinate system forms a triangle whose area is equal: (a) −16 (b) −8 (c) 8 (d) 16 **Question 5.** In symmetry to the origin of the coordinate system, the line has the equation (a) x + y − 4 = 0 (b) x + y + 4 = 0 (c) x − y + 4 = 0 (d) x − y − 4 = 0 **Question 6.** The function is (a) increasing (b) decreasing (c) constant (d) non-monotonic **Question 7.** The function takes values greater than 4 for its arguments (a) greater than 0 (b) greater than 8 (c) less than 0 (d) less than −4 **Question 8.** The line perpendicular to the function graph is: (a) y = 4x (b) y = − 1/4 x (c) y = −x (d) y = x	**Question 1.** The function graph intersects at two points (a) both axes of the coordinate system (b) each axis of the coordinate system (c) only X axis (d) only Y axis **Question 3.** The point with coordinates 0, 4 is the point where the function intersects (a) only X axis (b) only Y axis (c) both axes of the coordinate system (d) does not intersect any of the axes of the coordinate system **Question 4.** The point of intersection with the X axis has coordinates (a) −4, 0 (b) 4, 0 (c) 0, −4 (d) 0, 4 **Question 5.** The zero of the function is (a) 0 (b) 1 (c) 4 (d) −4 **Question 6.** The function takes non-positive values for x belonging (a) to the open range from −4 to infinity (b) to the right side closed range from minus infinity to −4 (c) to a left closed range from −4 to infinity (d) to the open range from minus infinity to −4 **Question 7.** The slope value of the functions is: (a) 1 (b) 4 (c) 0 (d) −4 **Question 8.** Indicate the false sentence: (a) the graph of the function passes through the I, II, and III quadrant of the coordinate system (b) function is decreasing (c) function is increasing (d) function takes negative values for arguments less than −4. **Question 9.** The graphs of the function with the line y = −x + 4 (a) are parallel but do not coincide (b) they intersect at one point but are not perpendicular. (c) they intersect at one point and are perpendicular. (d) coincide **Question 10.** The tangent of the angle of the function graph to the positive part of the OX axis is equal to (a) −4 (b) 0 (c) 1 (d) 4 **Question 11.** Equation of a line in the standard form: (a) x + y − 4 = 0 (b) x + y + 4 = 0 (c) x − y + 4 = 0 (d) x − y − 4 = 0 **Question 12.** In symmetry to the Y axis, the line has the equation: (a) x + y + 4 = 0 (b) x − y + 4 = 0 (c) x − y − 4 = 0 (d) x + y − 4 = 0	**Question 1.** The graph of the function intersects both axes of the coordinate system (a) in one point (b) in two points (c) in three points (d) does not intersect any axis **Question 2.** The point of intersection with the Y axis has coordinates (a) 0, 4 (b) 4, 0 (c) 0, −4 (d) −4, 0 **Question 3.** The value of the coefficient b in the function is: (a) −4 (b) 4 (c) 0 (d) 1 **Question 4.** The point of intersection with the X axis has coordinates: (a) 4, 0 (b) −4, 0 (c) 0, 4 (d) −4, 4 **Question 4.** The point of intersection with the X axis has coordinates (a) 4, 0 (b) −4, 0 (c) 0, 4 (d) −4, 4 **Question 5.** The zero of the function is (a) −4 (b) 0 (c) 1 (d) 4 **Question 6.** The function takes positive values for (a) x > 4 (b) x > 0 (c) x > − 4 (d) x < − 4 **Question 7.** The slope value of the functions is (a) 4 (b) −4 (c) 1 (d) 0 **Question 8.** Indicate the true sentence (a) The function decreases in the whole domain. (b) The function decreases for x < −4. (c) The function increases in the whole domain. (d) The function increases for x > 4 **Question 9.** Select a line parallel to the given straight line (a) y = 4x + 1 (b) y = − 4x + 16 (c) y = x + 6 (d) y = − x + 4 **Question 10.** The line perpendicular to the function graph is (a) y = x − 4 (b) y = x − 1/4 (c) y = − x + 5 (d) y = − 1/4 x + 1 **Question 11.** The tangent of the angle of the function graph to the positive part of the OX axis is equal to (a) −4 (b) 4 (c) −1 (d) 1 **Question 12.** Equation of a line in the standard form (a) x + y + 4 = 0 (b) x − y + 4 = 0 (c) x − y − 4 = 0 (d) x + y − 4 = 0 **Question 13.** In symmetry to the X axis, the line has the equation (a) x + y + 4 = 0 (b) x − y + 4 = 0 (c) x − y − 4 = 0 (d) x + y − 4 = 0 **Question 14.** For the argument −5, the function takes the value (a) −9 (b) −1 (c) 1 (d) 4
